# Facile Preparation and Characterization of Short-Fiber and Talc Reinforced Poly(Lactic Acid) Hybrid Composite with In Situ Reactive Compatibilizers

**DOI:** 10.3390/ma11071183

**Published:** 2018-07-10

**Authors:** Phornwalan Nanthananon, Manus Seadan, Sommai Pivsa-Art, Hiroyuki Hamada, Supakij Suttiruengwong

**Affiliations:** 1 Department of Materials Science and Engineering, Faculty of Engineering and Industrial Technology, Silpakorn University, Nakhon Pathom 73000, Thailand; p.nanthananon@hotmail.com; 2 Department of Physics, Faculty of Science, Silpakorn University, Nakhon Pathom 73000, Thailand; manus_sc.su@hotmail.com; 3 Department of Material and Metallurgical Engineering, Faculty of Engineering, Rajamangala University of Technology Thanyaburi, Pathumthani 12110, Thailand; sommai.p@en.rmutt.ac.th; 4 Kyoto Institute of Technology, Matsugasaki, Sakyo-ku, Kyoto City 606-8585, Japan; hhamada10294@gmail.com

**Keywords:** reactive hybrid composite, in situ reactive compatibilizer, facile preparation, poly(lactic acid) (PLA), one-step twin-screw extrusion

## Abstract

Hybrid composites of fillers and/or fibers reinforced polymer was generally produced by masterbatch dilution technique. In this work, the simplified preparation was introduced for the large volume production of 30 wt % short-fiber and talcum reinforced polymer hybrid composite by direct feeding into twin-screw extruder. Multifunctional epoxide-based terpolymer and/or maleic anhydride were selected as in situ reactive compatibilizers. The influence of fiber and talcum ratios and in situ reactive compatibilizers on mechanical, dynamic mechanical, morphological and thermal properties of hybrid composites were investigated. The morphological results showed the strong interfacial adhesion between fiber or talcum and Poly(lactic acid) (PLA) matrix due to a better compatibility by reaction of in situ compatibilizer. The reactive PLA hybrid composite showed the higher tensile strength and the elongation at break than non-compatibilized hybrid composite without sacrificing the tensile modulus. Upon increasing the talcum contents, the modulus and storage modulus of hybrid composites were also increased while the tensile strength and elongation at break were slightly decreased compared to PLA/fiber composite. Talcum was able to induce the crystallization of PLA hybrid composites.

## 1. Introduction

Hybrid composite is a system which contains two or more different types of reinforcing materials incorporated into a single matrix, for example, fillers and/or fibers reinforcement [1,2,3,4,5,6,7,8], or one type of reinforcing material presented in a blend of different matrices [3], or both approaches combined [9]. The main advantage of using hybrid composite could be supplemented with the lack of the other fillers, thus causing a balance in terms of cost and performance of composite materials [9]. The particles and short-fibers can be directly incorporated into thermoplastics by conventional processes, such as extrusion compounding and injection molding [1]. The effects of particle fillers and fibers on composite materials were investigated using a number of techniques, because they affected the mechanical properties of the polymer matrix [1,2,8]. However, improved performance of the hybrid composites was needed to ensure more use of renewable resources and sustainability.

Poly(lactic acid) (PLA) has extensively been explored for various applications due to its large-scale commercial availability, renewability and biodegradability. There are, however, few works carried out using natural fibers and particles reinforced PLA hybrid composites, for instance, PLA/newspaper fibers/talc [4], PLA/cellulose fiber/montmorillonite [7], PLA/cellulose fiber/nano calcium carbonate [7], and PLA/cellulose fiber/clay [6]. In our previous work on PLA biocomposites, the natural fiber from Bleach Eucalyptus Kraft Pulps (BEKP) was used as a reinforcing agent for PLA biocomposites due to its cost-effectiveness, whiteness, light weight, high purity of cellulose, high aspect ratio (40–50) and uniform diameter [10]. The addition of inorganic particle to polymer effectively enhanced the mechanical properties of the polymer matrix [1]. The addition of talcum (talc) as a filled particle in thermoplastics is a common practice [4,11,12,13] for promoting the performance in term of the improvement of mechanical and thermal properties of plastics with cost-effectiveness [1,13]. Ease of processing is also possible with the addition of talc.

An important concern for composite systems is the poor interaction between the polymer matrix and natural fibers and talc because a polymer matrix is hydrophobic while natural fiber and talc are naturally hydrophilic [14]. The incompatibility between the polymer matrix and the natural fibers or talc often reduces the capability of fillers and, hence, limits their practical usage [4,14]. Coupling agents are usually used to improve bonding between the filler and the thermoplastic by modifying their interfacial regions. Organo-functional silane is the most common coupling agent for modifying the surface of fillers [4,14]. In the case of surface treatment for a polymer matrix, maleic anhydride treatment was usually achieved though graft reactions [15,16,17]. Barletta and colleagues modified microlamellar talc by functionalization via hydrolysis and condensation reaction of its surface hydroxyl groups with different kinds of organosilanes (glycidyl, amino and isocyanate groups) in order to potentially be able to react with the -OH terminal groups of the PLA matrix [12]. In addition, they also investigated PLA–talc biocomposites that involve two compatibilizing agents, maleic anhydride (MA) and glycidyl methacrylate (GMA)-grafted PLA [12]. However, such methods were time-consuming and difficult to scale up for large industrial production. Based on these aspects, in situ reactive processing can be introduced and applied for hybrid composite processing, allowing the possibility of increasing the interfacial adhesion with a cost-effectiveness in terms of the manufacturing technique [10]. Some of the current in situ compatibilizers used for each biopolymer blends have been investigated, such as Methylene diphenyl diisocyanate (MDI) used in Poly(lactic acid)/Polybutylene succinate [18] and Poly(l-lactic acid)/Polycaprolactone [19], Dicumyl peroxide (DCP) used in Poly(l-lactic acid)/Polybutylene succinate [20] and PHB/Polybutylene succinate [21], Joncryl used in Poly(lactic acid)/PBSA [22], Poly(lactic acid)/Polypropylene carbonate [23] and Poly(l-lactic acid)/PBAT [24], PLA-g-GMA (glycidyl methacrylate grafted Poly(lactic acid) used in PLA/Starch [25], PLA-g-MA (Maleic anhydride-grafted Poly(lactic acid)) used in Poly(lactic acid)/Polycaprolactone [26], Poly(lactic acid)/Starch [27] and Poly(l-lactic acid)/Polybutylene adipate terephthalate [28]. From our previous work, different types of reactive agents including multifunctional epoxide-based reactive terpolymer (CEGMA, EAGMA) and maleic anhydride-based reactive terpolymer (EAMAH) were introduced as in situ reactive compatibilizer in the processing of PLA biocomposites [10]. It was found that CEGMA was the most effective compatibilizer for PLA and natural fiber biocomposite, as the optimal mechanical properties could be realized [10]. Hao and colleagues studied the compatibilization between PLA and sisal fiber (SF) biocomposites using an epoxy-functionalized terpolymer elastomer (EGMA) as an in situ compatibilizer. They found that the addition of EGMA into PLA/SF composites, not only enhanced the interfacial compatibility of fiber and PLA matrix, but also improved the toughness of composites without much deterioration of the tensile strength [29].

To the best of our knowledge, there have not yet been reports about in situ reactive compatibilization of natural fibers and particle-reinforced PLA hybrid composites. Another challenge was the processing of the hybrid composite, because it was difficult to load a high content of fillers with polymers into a conventional twin-screw extruder without side-feeding due to the different densities of the individual materials. The existing solution is masterbatch preparation, but this is a time and energy-consuming process and is difficult to scale up for large volumes. Therefore, the aim of this work is to introduce a simplified method for producing PLA hybrid composites consisting of 30 wt % of short-fiber and talc using one-step twin-screw extrusion using in situ reactive compatibilization. Multifunctional epoxide-based reactive terpolymer (CEGMA) was selected as an in situ single compatibilizer and incorporated with maleic anhydride (MAH) as multiple compatibilizers. The influence of the filler ratios (talc and fiber) and the in situ reactive compatibilizer on the morphological mechanical, dynamic mechanical, and thermal properties of hybrid composites were investigated.

## 2. Materials and Methods

### 2.1. Materials

Poly(lactic acid) (PLA), 3052D injection grade was purchased from NatureWorks^®^ LLC (Minnetonka, MN, USA). Bleach Eucalyptus Kraft Pulps (0.538 mm in average length and 13.90 μm in diameter) were kindly supplied by SCG Packaging PLC., Ratchaburi, Thailand. Talcum, 1250 grade was purchased from Thai Poly Chemicals Co., Ltd., Samut Sakhon, Thailand. An epoxy-based chain extender Joncryl^®^ ADR-4368F (designated as CEGMA) was obtained from BASF Chemical Co., Ltd., Bangkok, Thailand. Maleic anhydride, 99.0% purity, (designated as MAH) was purchased from Sigma-Aldrich, Bangkok, Thailand. Peroxide (Perkadox^®^ 14-40B-pd) was purchased from AkzoNobel (Bangkok, Thailand). Ethanol and chloroform were purchased from Better Syndicate Co., Ltd., Bangkok, Thailand.

### 2.2. Preparation of PLA/NF Particles

The dried Bleach Eucalyptus Kraft Pulp (BEKP) was ground to obtain a natural fiber (NF) using a high-speed grinder. NF was dried at 80 °C in a vacuum oven for 24 h. To allow for ease of fiber feeding, the density of NF, PLA was firstly dissolved in chloroform using a mechanical stirrer at 60 °C for 30 min and then 60 wt % of NF was added to the solution containing 40 wt % of PLA. The PLA/NF mixed suspension was evaporated to remove chloroform solvent at room temperature for 1 h and then dried at 80 °C hot air oven for 6 h. Finally, the dried mixed PLA/NF was crushed into particles.

### 2.3. Facile Preparation of Hybrid Fillers between Natural Fiber and Talc

The preparation of the hybrid of NF and talc was divided into two routes. In the first route, talc was dispersed in ethanol and then NF, with and without CEGMA, was added into the talc suspension under NF-to-talc ratios of 2:1, 1:1, and 1:2. In the second route, MAH was firstly dissolved in ethanol and then talc was dispersed in the MAH solution under stirring. After that, NF followed by CEGMA were added into the mixed suspension, respectively, using the same fillers ratios of the first case, under constant stirring. The mixed suspension of each case was continuously stirred for 2 min using an electric mixer at room temperature to obtain the suspended hybrid fillers of NF and talc. After that, the mixed suspensions were dried at 80 °C in a hot air oven for 24 h to remove ethanol. Finally, hybrid fillers, with and without deposited reactive compatibilizers, were obtained as particles ranging between 2–7 mm in diameter.

### 2.4. Facile Preparation of PLA Composites and PLA/NF/Talc Hybrid Composites

**Figure 1:**
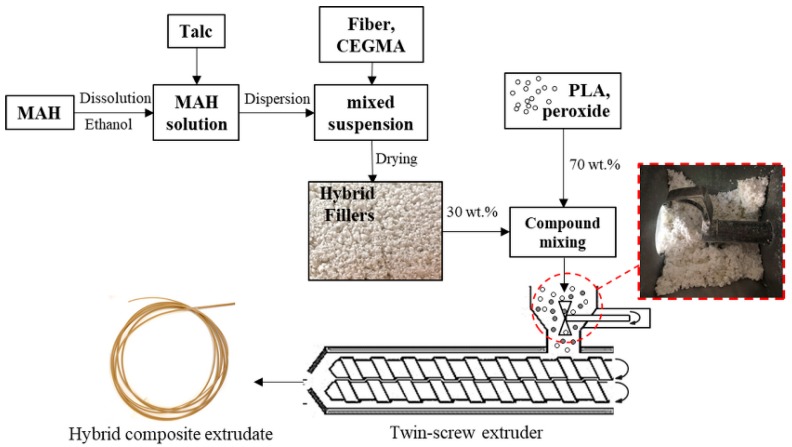
Schematic representation of the simplified preparation of hybrid fillers and extrusion compounding process of the reactive hybrid composites.

PLA resin was dried at 60 °C in a vacuum oven for 12 h. The various compositions of hybrid fillers were easily loaded together with PLA into a hopper, with and without peroxide, as listed in Table 1. PLA, NF and/or talc were melt-blended using a twin-screw extruder (SHJ-26, L/D = 40, ENMACH Co., Ltd., Nonthaburi, Thailand). The temperature profile of the twin-screw extruder was set from the feed zone to die as 130, 140, 150, 170, 180, 190, 190, 180, 170, and 170 °C under a screw speed of 150 rpm. The scheme of the extrusion compounding process of the reactive hybrid composites was shown in Figure 1. After that, hybrid composite extrudates were cut to produce pellets and then dried for 24 h at 60 °C in a hot air oven.

### 2.5. Tensile Specimens Preparation

After drying, the hybrid composites and neat PLA pellets were placed in an injection molding machine (SM-22, Battenfeld, Kottingbrunn, Austria) in order to produce tensile test specimens. The processing conditions for the injection molding are summarized as follows: Nozzle temperature and cooling time were 190 °C and 42 s, respectively.

### 2.6. Testing and Characterization

#### 2.6.1. Mechanical Properties

Mechanical properties were carried out by using a universal testing machine (Instron 5969, Norwood, MA, USA) according to ASTM D638 [30], specimen type I, and a 5-KN load cell. The testing was conducted under ambient conditions using a cross-head speed of 5 mm/min. All reported values were obtained as averages of five specimens.

#### 2.6.2. Morphological Observation

The tensile fracture surface morphology of the PLA composite and hybrid composite specimens were observed using a Field Emission Scanning Electron Microscope (FE-SEM) (TESCAN MIRA3 LMH Schottky, Brno, Czech Republic) at an accelerating voltage of 5 kV. Prior to observations, the samples were sputter coated with a thin layer of gold to avoid charging.

#### 2.6.3. Dynamic-Mechanical Thermal Analysis (DMTA)

To produce DMTA specimens, the PLA composite and hybrid composite were pre-heated for 4 min and then compressed at 190 °C and 1000 psi pressure for 1 min. After that, the samples were laser-cut to dimensions of 10 × 1.16 × 40 mm^3^. Dynamic-mechanical thermal properties of PLA composites and the hybrid composite were examined using an ANTON PAAR, modular compact rheometer (MCR302, Graz, Austria) equipped with rectangular fixture (SRF) holders. A temperature sweep was heated from 30 to 110 °C at a heating rate of 3 °C/min under the DMTA torsion mode at a frequency of 1 Hz.

#### 2.6.4. Differential Scanning Calorimetry (DSC)

Differential scanning calorimetry (DSC) of samples were carried out on a DSC1 STAR instrument (METTLER TOLEDO, Greifensee, Switzerland) under a nitrogen atmosphere. The samples were first heated from 30 to 200 °C at a rate of 10 °C/min and held on for 1 min. The samples were then cooled down to 30 °C at the rate of 10 °C/min and reheated to 200 °C at the same conditions. The degree of crystallinity (*X*_c_) was calculated according to the following equation [31]:

(1) $$Xc\;\left(\%\right)=\;\frac{∆H_{m}\;-\;∆H_{\mathrm{cc}}}{(\omega_{\mathrm{PLA}})∆H_{m}^{o}}\;\times \;100,$$

where ΔH_m_ was the melting enthalpy and ΔH_cc_ was the cold crystallization enthalpy measured from the thermogram peak, $\omega_{\mathrm{PLA}}$ was the weight fraction of PLA, and $∆H_{m}^{o}$ was the ideal melting enthalpy for completely crystalline PLA of 93.6 J/g [32].

## 3. Results

### 3.1. Mechanical Properties

**Figure 2:**
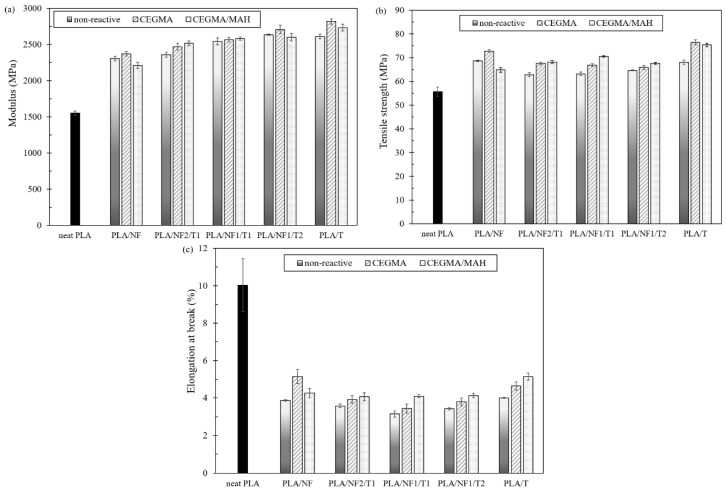
(**a**) Modulus; (**b**) tensile strength and (**c**) elongation at break of PLA hybrid composites at different fillers ratios, with and without various reactive compatibilizers.

Modulus, tensile strength and elongation at break of neat PLA and PLA/NF/T hybrid composites at different ratios, with and without various reactive compatibilizers, are shown in Figure 2. All PLA hybrid composites showed a much higher modulus and lower elongation at break than that of neat PLA, indicating the enhanced stiffness due to the presence of fillers [33,34]. In addition, the tensile strength of all PLA hybrid composites increased when compared to that of neat PLA, caused by the reinforcing effect of the fillers [35]. As the talc content increased for non-reactive hybrid composites, the modulus tended to increase slightly by 2%, 10% and 14% for NF2/T1, NF1/T1 and NF1/T2 ratios, respectively, compared to PLA-composite-added fiber. These results indicated a more enhanced stiffness of PLA hybrid composites due to the presence of talc. PLA-composite-added talc showed an increase in the modulus, as reported by many researchers [36]. The tensile strength and elongation at break of the hybrid composites were decreased after increasing the talc content, resulting from the poor interfacial adhesion between the fillers and matrix; hence, the stress concentration under loading conditions [29].

The incorporation of reactive compatibilizers (CEGMA or CEGMA/MAH) into PLA/NF/T hybrid composites did not change the modulus significantly compared to non-compatibilized composite and hybrid composite, except at a higher fiber content. On the other hand, the presence of CEGMA/MAH in PLA hybrid composites showed a higher tensile strength than adding CEGMA, compared to their non-compatibilized hybrid composites. In the case of the PLA/T composite, it showed the highest tensile strength, regardless of whether CEGMA or CEGMA/MAH was added. This implied a better interfacial adhesion between the PLA matrix and fiber or talc with the addition of reactive compatibilizer, which caused a better stress transfer from the PLA matrix to the fillers. Furthermore, the incorporation of both reactive compatibilizers demonstrated an improvement in the elongation at break of the hybrid composites. The presence of CEGMA/MAH showed the highest elongation at break of about 14%, 30%, and 21% for PLA/NF2/T1, PLA/NF1/T1 and PLA/NF1/T2, respectively, when compared to the non-compatibilized hybrid composites. This indicated the synergistic effect of the compatibilizer between CEGMA and MAH.

### 3.2. Morphological Observation

**Figure 3:**
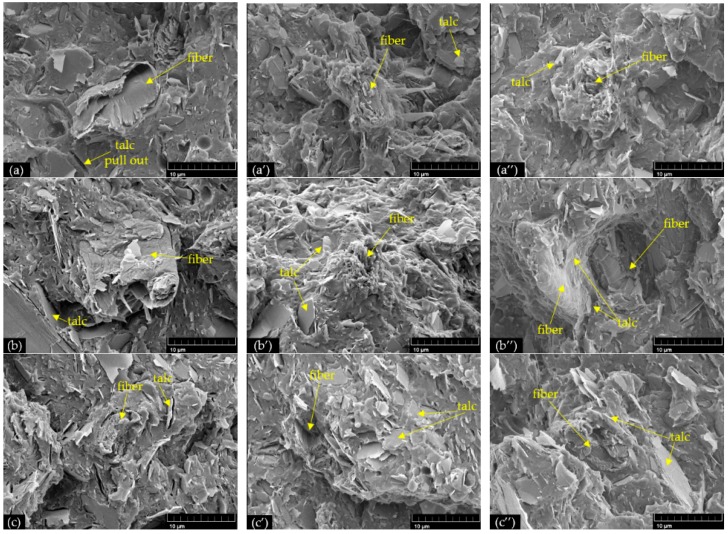
Tensile fracture surfaces of PLA hybrid composites with and without reactive compatibilizers: (**a**) PLA/NF2/T1; (**a’**) PLA/NF2/T1/CEGMA; (**a’’**) PLA/NF2/T1/CEGMA/MAH; (**b**) PLA/NF1/T1; (**b’**) PLA/NF1/T1/CEGMA; (**b’’**) PLA/NF1/T1/CEGMA/MAH; (**c**) PLA/NF1/T2; (**c’**) PLA/NF1/T2/CEGMA; (**c’’**) PLA/NF1/T2/CEGMA/MAH.

The tensile fracture surfaces of PLA/NF/T hybrid composites at different filler ratios, with and without various reactive compatibilizers, were observed using FE-SEM, as shown in Figure 3. The different filler ratios did not affect the interfacial adhesion of hybrid composites. PLA hybrid composites without reactive compatibilizer, as illustrated in Figure 3a–c, exhibited voids between the PLA matrix and fiber or talc, and some pull-outs of talc because of the poor interfacial adhesion between the fillers and PLA matrix. In contrast, the incorporation of CEGMA and CEGMA/MAH in PLA hybrid composites, as shown in Figure 3a’–c’ and 3a’’–c’’, respectively, showed that fiber was broken and torn out on the fracture surface without voids between the fiber and matrix being observed. In addition, they PLA matrix tightly adhered to both fiber and talc surfaces. This indicated that the interfacial adhesion of the PLA matrix, with both fiber and talc in hybrid composites, was considerably improved by adding multifunctional epoxide compatibilizer with and without MAH. 

### 3.3. Dynamic-Mechanical Thermal Analysis (DMTA)

**Figure 4:**
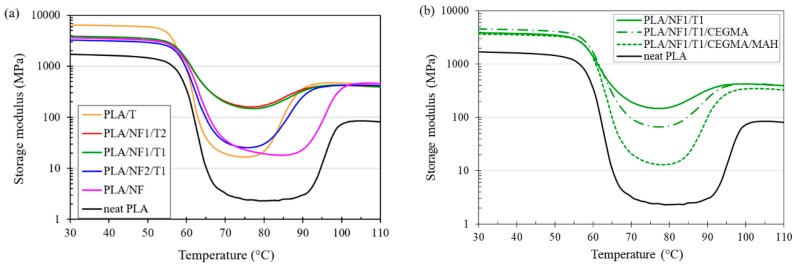
Plot of storage modulus against temperature of PLA hybrid composites at: (**a**) different fillers ratios; (**b**) with and without various reactive compatibilizers.

**Figure 5:**
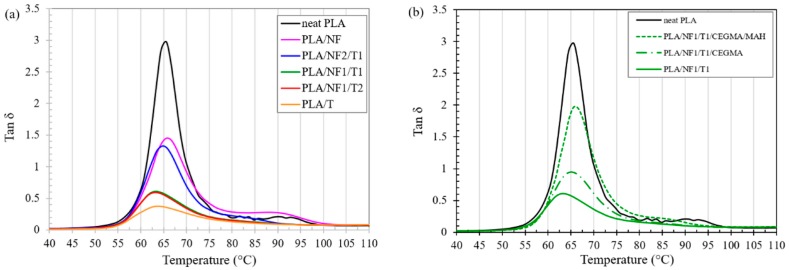
Plot of tan δ against temperature of PLA hybrid composites at: (**a**) different fillers ratios; (**b**) with and without various reactive compatibilizers.

The dependence of the storage modulus versus the temperature of the PLA hybrid composites at different fillers ratios, with and without reactive compatibilizers, is depicted in Figure 4. The storage moduli of all hybrid composites and single filler composites were much higher than that of neat PLA in the glassy region (30–50 °C), which is obviously seen in PLA/T composite. It was noticed that PLA hybrid composites and neat PLA started softening above glass transition temperatures of around 60 °C. It is clear from Figure 4a that the storage moduli of PLA/NF1/T1 and PLA/NF1/T2 hybrid composites dropped slightly in the rubbery region and then leveled off (70–80 °C), indicating an improvement in the heat distortion temperature (HDT) [37] of the hybrid composites compared to neat PLA and single filler composites. In the rubbery region, the storage moduli of hybrid composites at NF2/T1, NF1/T1 and NF1/T2 ratios were increased by 10, 64 and 64 times compared to that of neat PLA, and increased by 2, 10 and 10 times compared to that of the single filler composite. The remarkably-higher storage modulus in the rubbery region of the PLA/NF1/T1 and PLA/NF1/T2 hybrid composites was due to the effect of hybridization, which could not happen in the single filler composites. For the addition of CEGMA or CEGM/MAH, it can be seen in Figure 4b that the storage modulus of the hybrid composite was decreased. For the addition of CEGMA or CEGM/MAH, it was found from Figure 4b that the storage modulus of the hybrid composite was decreased. It was determined that the storage modulus of neat PLA and PLA/NF started to increase again after 90 °C, which was relevant to its recrystallization [38]. The storage moduli of other PLA hybrid composites, with and without both reactive compatibilizers, started to increase again at around 80 °C, indicating a faster crystallization than that of neat PLA and PLA/NF [37]. Figure 5 shows a plot of tan *δ* (ratio of the loss modulus to the storage modulus) versus temperature of PLA hybrid composites, with and without reactive compatibilizers. Figure 5a illustrates that the tan *δ* peak height of the PLA/NF/T hybrid composite was lower than that of only the PLA/NF composite, and was much lower than that of neat PLA. PLA-composite-added talc showed the lowest tan *δ* peak height. This could describe that the mobility of the PLA chain was restricted by the addition of natural fiber [4], and, especially, by adding talc because it was a platy filler [39]. Figure 5b shows that the tan *δ* peak height of the PLA/NF1/T1 hybrid composite with CEGMA/MAH was higher than that incorporated with CEGMA, and much higher than those of the non-compatibilized hybrid composites.

### 3.4. Differential Scanning Calorimetry (DSC)

**Figure 6:**
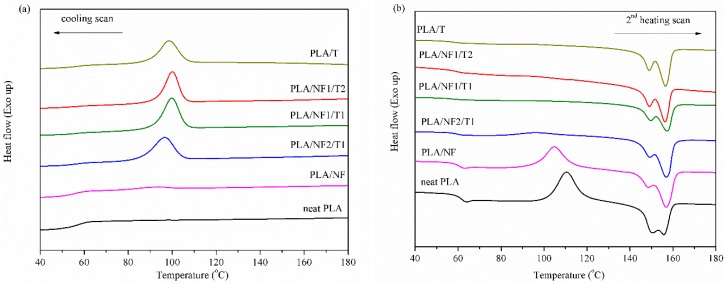
DSC thermograms of PLA hybrid composites at different filler ratios: (**a**) cooling scan and (**b**) second heating scan.

**Figure 7:**
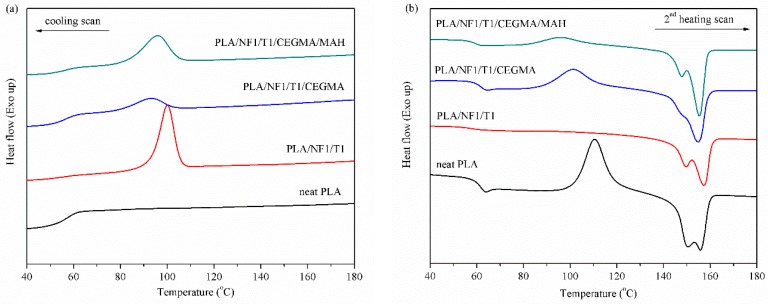
DSC thermograms of PLA hybrid composites with and without reactive compatibilizers at NF1/T1 ratio: (**a**) cooling scan and; (**b**) second heating scan.

**Figure 8:**
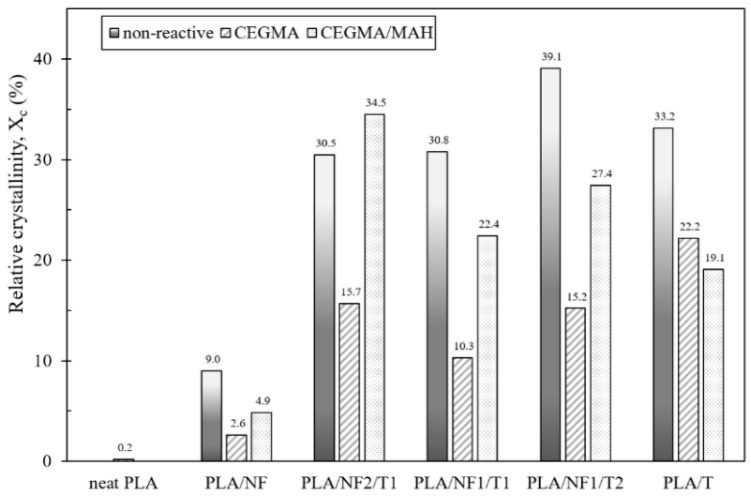
Degree of crystallinity (*X*_c_) of PLA hybrid composites at different filler ratios, with and without various reactive compatibilizers.

DSC thermograms of neat PLA and PLA hybrid composites at different filler ratios, without reactive compatibilizers, are shown in Figure 6. As the talc ratio increased in the PLA hybrid composites, a large crystallization peak and higher crystallization temperature (compared to that of neat PLA in the cooling scan) were observed. In addition, it was noticed that the peak of the cold crystallization temperature for PLANF1/T1 and PLA/NF1/T2 almost disappeared in the heating scan because the crystallization was completed during a cooling step. In contrast, the cold crystallization peak of neat PLA and PLA/NF appeared at about 110 and 105 °C, respectively, in the heating scan (Figure 7b), due to the low crystallization ability [32]. As is well known, talc is able to induce the crystallization of PLA [12,31,37,40]; hence, more talc concentrations added caused more crystallinity of PLA in hybrid composites as seen in Figure 8. In the case of short-fiber introduction, a slight improvement in crystallinity of the PLA/NF composite compared to that of neat PLA was ddmonstrated. This effect could be attributed to the fact that a small and short fiber acted as a nucleating site for the crystallization of PLA around the surface of the fiber [41]. The effect of the incorporation of various reactive compatibilizers on crystallization of the compatibilized PLA hybrid composites is shown in Figure 7. It was found that the crystallization and crystallinity of the PLA hybrid composites tended to decrease compared to the non-compatibilized hybrid composites, which are clearly illustrated in Figure 7 and Figure 8. However, the cold crystallization on the set temperature of the compatibilized PLA hybrid composites was lower than that of neat PLA, suggesting that its overall cold crystallization rate was faster [42].

## 4. Discussion

The large improvement in tensile strength could be described by the better interfacial adhesion between the fillers and PLA matrix when the multifunctional epoxide-based compatibilizer, with and without MAH, was added. This led to a decrease in the stress concentration and a better stress transfer from the PLA matrix to fiber or talc. From the morphology results, a significant improvement in the interfacial adhesion between PLA matrix and fibers or talc in hybrid composites was found after adding multifunctional epoxide terpolymer. This phenomenon could be attributed to the reaction between the presence of the epoxide groups in the multifunctional epoxide terpolymer and the hydroxyl groups in the fibers or talc structure and the end group of the PLA molecular chain during the melt-blending process, as proved by FT-IR analysis of the extracted fibers (reported elsewhere) [10,29]. Huda and colleagues reported that the -OH in talc structure could react with MA- and GMA-grafted-PLA, which led to good compatibilization of PLA/talc compounds with good processability [12]. Generally, MAH could be grafted onto PLA chains via free radical reactions using peroxide in the melt state. From these results and reports, it could be implied that the addition of CEGMA and/or MAH with peroxide could reactively bond with the hydroxyl group on fiber, talc and the chain-end of PLA via an in situ melting-process and, thus, improved the interfacial adhesion. In addition, crystallization of the PLA hybrid composite, incorporating reactive compatibilizers, tended to decrease compared to non-compatibilized hybrid in the cooling step. This might be due to the lower molecular chain mobility of PLA in the presence of CEGMA, which acted as a chain extender and led to an increase in PLA molecular weight. However, the presence of CEGMA with MAH and peroxide in the PLA/NF/T hybrid composite showed a higher crystallinity than adding CEGMA without MAH/peroxide. One possible explanation was that PLA-grafted-MAH using peroxide had a low molecular weight and could act as a lubricant on the PLA matrix, which enhanced PLA chain mobility and led to increasing the crystallization activity [36], more than adding only CEGMA. More studies on the interfacial reaction and microstructure of PLA/fiber/talc hybrid composites incorporating CEGMA and/or MAH should be conducted in the future in order to understand the chemical reactions of the reactive compatibilizers.

## 5. Conclusions

The short-fiber and talc reinforced PLA hybrid composites, with and without reactive compatibilizers, were successfully prepared using one-step direct feeding via a twin-screw extruder. For the influence of the filler ratios as the talc ratio increased, the modulus of the hybrid composites was increased while the tensile strength and elongation at break were decreased compared to PLA/NF. In the rubbery region, the storage modulus of hybrid composites was increased by 10 times for the NF1/T1 and NF1/T2 ratios compared to PLA/NF3/T0. Talc was able to induce the crystallization of PLA hybrid composites. The interfacial adhesion between fibers or talc and the PLA matrix was significantly improved when the in situ reactive compatibilizers (CEGMA or CEGMA/MAH) were incorporated. The largest improvements in tensile strength were 8% and 11% for the addition of CEGMA and CEGMA/MAH, respectively, when compared to the non-compatibilized hybrid composites. In addition, the elongation at break showed the highest increase, by 10% and 30% for the addition of CEGMA and CEGMA/MAH, respectively, compared to non-compatibilized hybrid composites. The reaction and microstructure of PLA/fiber/talc hybrid composites incorporated with CEGMA and/or MAH with peroxide will be conducted by our group in the near future in order to further understand the role of each reactive compound.

## Figures and Tables

**Table 1:** List of material compositions for extrusion compounding.

Sample Codes	PLA (g)	Natural Fiber (g)	Talc (g)	CEGMA (phr)	MAH (phr)	Peroxide (phr)
neat PLA	100					
PLA/NF	70	30	0			
PLA/NF2/T1	70	20	10			
PLA/NF1/T1	70	15	15			
PLA/NF1/T2	70	10	20			
PLA/T	70	0	30			
PLA/NF/CEGMA	70	30	0	1.5		
PLA/NF2/T1/CEGMA	70	20	10	1.5		
PLA/NF1/T1/CEGMA	70	15	15	1.5		
PLA/NF1/T2/CEGMA	70	10	20	1.5		
PLA/T/CEGMA	70	0	30	1.5		
PLA/NF/CEGMA/MAH	70	30	0	1.5	1.5	0.1
PLA/NF2/T1/CEGMA/MAH	70	20	10	1.5	1.5	0.1
PLA/NF1/T1/CEGMA/MAH	70	15	15	1.5	1.5	0.1
PLA/NF1/T2/CEGMA/MAH	70	10	20	1.5	1.5	0.1
PLA/T/CEGMA/MAH	70	20	10	1.5	1.5	0.1
